# Enzymatic Deacidification and Aroma Characteristics Analysis of Rapeseed Oil Using Self-Made Immobilized Lipase CALB@MCM-41-C_8_

**DOI:** 10.3390/foods13162539

**Published:** 2024-08-14

**Authors:** Zhonghui Liu, Tieliang Liu, Run Liu, Qi Zhou, Yandaizi Zhou, Yi Zhang, Mingming Zheng

**Affiliations:** 1 Wuhan Institute of Technology, School of Chemical Engineering and Pharmacy, Key Laboratory of Green Chemical Process of Ministry of Education, Hubei Key Laboratory of Novel Reactor and Green Chemical Technology, Wuhan 430205, China; liuzhonghui0919@163.com; 2 Oil Crops Research Institute, Chinese Academy of Agricultural Sciences, Hubei Key Laboratory of Lipid Chemistry and Nutrition, Hubei Hongshan Laboratory, Key Laboratory of Oilseeds Processing, Ministry of Agriculture, Wuhan 430062, China; ltl13164666251@163.com (T.L.); liurun5764@163.com (R.L.); zhouqi01@caas.cn (Q.Z.); zhengmingming@caas.cn (M.Z.)

**Keywords:** rapeseed oil, immobilized lipase, enzymatic deacidification, diacylglycerols, antioxidant stability, aroma characteristics

## Abstract

Rapeseed oil is a widely consumed edible oil that contains varieties of beneficial micronutrients such as tocopherols and phytosterols; however, the high acid value due to increased free fatty acid can imperil the oil quality and safety. This paper proposed the enzymatic deacidification for high-acid rapeseed oil and simultaneous production of functional diacylglycerols (DAGs) catalyzed by self-made immobilized lipase CALB@MCM-41-C_8_. The results indicate that the carrier of molecular sieve MCM-41 exhibited a sufficient surface area of 1439.9 m^2^/g and a proper pore size of 3.5 nm, promoting the immobilization of lipase CLAB. Under the optimal reaction conditions, the acid value of rapeseed oil was largely decreased from 15.3 mg KOH/g to 1.7 mg KOH/g within 3 h, while DAG content was increased from 1.2% to 40.2%. The antioxidant stability of rapeseed oil was also increased from 4.3 h to 7.6 h after enzymatic deacidification. Besides, the deacidified rapeseed oil exhibited fatty, bitter almond aromas, compared to the picked-vegetable, spicy, and pungent aromas for high-acid oil. Finally, the catalytic stability and applicability of CALB@MCM-41-C_8_ was validated, thus demonstrating the great potential of CALB@MCM-41-C_8_ in green refining of edible oils and sustainable synthesis of functional lipids.

## 1. Introduction

Rapeseed oil, commonly used edible oil, is ranked as the second largest oil crop with an annual production of approximately 12 million tons [1,2]. Rapeseed oil is enriched in numerous bioactive components, including tocopherols, phytosterols, and squalene that are beneficial in preventing coronary heart disease, diabetes, hypertension, etc. [3,4,5,6]. However, the high temperature, light exposure, and humidity air during oil extraction, storage, and processing can accelerate the hydrolysis of triglycerides, causing the generation of free fatty acids (FFAs) which is reflected by the increased acid values and rancid smell [7,8]. The rancid oils not only diminish their economic values but also pose threats to public health [9,10]. Currently, high-acid oils are only used as cheap raw materials for industrial production, or even simply discarded to cause huge resource waste and environmental pollution [11,12]. Hence, deacidification is a primary and essential step to improve the quality and applicability of edible oils.

Traditional oil deacidification primarily relies on alkali refining, which uses sodium hydroxide to saponify free fatty acids and reduce the acid value of crude oil [13]. However, a large amount of crude oil is entrapped in the soap micelles and removed with the soap during centrifugation, resulting in the fact that the loss of neutral oil and micronutrients such as tocopherol, gamma oryzanol, and phytosterols. Moreover, a large amount of wastewater will be generated, which will cause serious pollution to the environment. Other deacidification methods include distillation and solvent extraction, where distillation can be inefficient due to high temperatures and energy consumption, and solvent extraction may pose risks of solvent residue [14,15,16,17,18,19]. Furthermore, oils extracted and deacidified by solvents require additional separation and purification, consuming more resources. Hence, a cleaner and greener approach to improve oil deacidification is highly required. 

Enzymatic deacidification relies on lipases to catalyze the esterification between free fatty acids (FFAs) and specific acyl acceptors such as glycerol, monoglycerides, and phytosterol to reduce the acid value of oils. Compared to chemical methods, the enzymatic approach not only reaches the oil deacidification in greener and cleaner ways but also simultaneously generates functional lipids such as monoglycerides (MAGs), diacylglycerol (DAG), and phytosterol esters (PEs) [20,21]. To improve the catalytic stability and reusability of free enzymes, researchers have attempted various enzyme immobilization techniques including physical adsorption, encapsulation, chemical cross-linking, and covalent to bind or restrict enzyme molecules to solid carriers [22,23]. Based on this concept, commercialized immobilized lipases such as Nozoyme 435, Lipozyme TL IM, and RM IM have been developed and widely used in oil processing [24,25,26]. However, the high cost of commercialized immobilized lipases is the main reason to suspend their industrial application. Moreover, the carriers including microporous resins or silica gels are easily deformed or fragile at extreme conditions with high temperatures, fierce agitation, and polar solvents [27]. Hence, the selection of robust carriers is the key step for the preparation of stable and efficient immobilized lipases. 

Recently, different porous solids such as porous carbon materials (PCMs), organic-inorganic hybrid porous material (KCS-2), hollow multihulled solids, and magnetic nanoparticles have been reported for enzyme immobilization [28,29,30,31]. However, these carriers are constrained by the long preparation period, complex preparation procedures, and high cost; therefore, these particles were only used for lab-scale research instead of for industrial application. Instead, molecular sieves are mainly prepared based on silica, alumina, and some metal oxides, with a specific surface area of up to 1000 m^2^/g, a pore volume of up to 2.5 cm^3^/g, and an average aperture between 2 and 50 nm [32]. Based on the different porous structures, the molecular sieves can be classified according to spatial structure into one-dimensional pore systems (MCM-41, SBA-15, FSM-16), three-dimensional pore systems (MCM-48), and partially disordered structures (MCF, CNS) [33]. The advantages such as corrosion resistance, high-temperature tolerance, and superior mechanical strength have led to the wide use of molecular sieves in biomedicine, food processing, and fine chemicals industry areas [34,35,36]. Specifically, mesoporous molecular sieves with pore sizes ranging from 2 to 10 nm make them appropriate carriers for enzyme immobilization [37]. For instance, Rui et al. [38] found that the immobilized acetylcholinesterase (AChE) exhibited 1.3 times higher catalytic activity than the free enzyme and retained 94% of its initial activity after 60 days of reuse. Lei et al. [39] reported that immobilized naringins based on the mesoporous molecular sieve of MCM-41 could facilitate 95% of debittering rates for grapefruit juice in 30 min. Thus, mesoporous molecular sieves have shown great potential in enzyme immobilization, which is applicable for the enzymatic refining of edible oils. 

This study prepared an efficient and robust immobilized lipase based on the hydrophobically modified mesoporous molecular sieve MCM-41, which was then used for the enzymatic deacidification of high-acid rapeseed oil. Here, the different mesoporous molecular sieves including MCM-21, MCM-41, MCM-49, and various lipases (i.e., CALB, AYS, PS, CSL, AY400SD) were investigated and screened. Then, the lipase immobilization conditions and the enzymatic reaction conditions including temperature, substrate molar ratio, and adding an amount of CALB@MCM-41-C_8_ 4 Å molecular sieve were optimized. Moreover, the catalytic stability and usability of CALB@MCM-41-C_8_ were also explored. The aroma characteristics and oil quality (i.e., the oxidation stability, and peroxide values) of rapeseed oils before and after enzymatic deacidification were compared. Overall, this paper provides an efficient route for rapeseed oil deacidification based on the self-made immobilized lipase CALB@MCM-41-C_8_.

## 2. Materials and Methods

### 2.1. Experimental Materials

Rapeseed oil is obtained from the Oil Crops Research Institute of the Chinese Academy of Agricultural Sciences. Different mesoporous molecular sieves including MCM-22, MCM-41, and MCM-48 were purchased from Jiangyin Nanda Synthetic Chemical Co., Ltd. (Jiangyin, China). *Candida* sp. lipase (CSL, lyophilized powder), Candida antarctica lipase B (CALB, lyophilized powder), free Candida cylindricalis (AYS and AY400SD, lyophilized powder), Pseudomonas cepacian lipase (PS, lyophilized powder) were obtained from Amano Enzyme Inc. (Nagoya, Japan). Bicinchoninic acid (BCA) Protein Assay Kit was obtained from Shanghai Yuanye Bio-Technology Co., Ltd. (Shanghai, China). Glycerol, oleic acid, isopropanol, ligroin (boiling point at 30–60 °C), n-hexane, phenolphthalein, 95% ethanol, n-octyltrichlorosilane and other chemicals were purchased from Aladdin Reagent Co., Ltd. (Shanghai, China). All reagents and solvents used in the experiments were of analytical and chromatographic grade or biological grade. Ultrapure water was purified using Milli-Q water by MilliQ apparatus (Millipore, Bedford, MA, USA).

### 2.2. Surface Functionalization of MCM-41 and Lipase Immobilization

To obtain a hydrophobic carrier for lipase immobilization, the surface of MCM-41 was treated through grafting modification with a silane coupling agent of C_8_ [40]. Firstly, 0.5 g of MCM-41 was mixed with 5 mL of n-hexane and 231 μL of n-octyltrichlorosilane at room temperature, which were stirred at the speed of 220 rpm for 40 min. Then, the well-mixed solutions were filtered using a Buchner funnel to collect the reacted solids, which were moved into a 60 °C drying oven (DHG-9030A, Shanghai Yiheng Scientific Instrument Co., Ltd., Shanghai, China) for 24 h. Finally, the dried solids, namely, MCM-41-C_8_, were stored in the dryer at room temperature for long-term use.

The immobilization of Candida antarctica lipase B (CALB) on MCM-41-C_8_ was prepared based on the method reported by Lai et al. [41]. To start, a certain amount of CALB was added into phosphate buffer (50 mM, pH 5–9) and stirred at 4 °C for 30 min to obtain the lipase solution. Then, 0.1 g of MCM-41-C_8_ was then prewetted with 200 μL of pure ethanol, followed by being immersed into 10 mL of prepared lipase solution and incubated in a shaking incubator (THZ-C, Suzhou Peiving Experimental Equipment Co.actorv, Suzhou, China) with the temperature of 30 °C and the shaking speed of 220 rpm for 40 min. Afterward, the whole contents were centrifuged using a refrigerated centrifuge (Allegra X-12, Beckman Coulter, Inc., Brea, CA, USA) at 8000 rpm for 10 min to obtain the supernatant, which was used to determine the amount of residual protein using a BCA protein assay. Finally, the precipitates of immobilized lipase CALB@MCM-41-C_8_ were collected and washed using phosphate buffer at least 3 times and freeze-dried in a vacuum freeze-drying machine (YTLG-18A, Shanghai Yetuo Technology Co., Ltd., Shanghai, China) to obtain the targeted CALB@MCM-41-C_8_. 

### 2.3. Characterization of MCM-41, MCM-41-C_8_, and CALB@MCM-41-C_8_

The surface morphologies and microscopic internal structures of the MCM-41 were observed using SEM (S-4800, Hitachi, Japan) and TEM (TECNAI G2 20S-TWIN, FEI Company, Hillsboro, OR, USA), respectively. The surface area and total pore volumes of MCM-41, MCM-41-C_8_, and CALB@MCM-41-C_8_ were measured with a computer-controlled nitrogen gas adsorption analyzer (ASAP2010, Micromeritics, Norcross, GA, USA) in the relative pressure range of 0.05–1.00 (specifically, the nitrogen partial pressure is nondimensionalized with the saturated vapor pressure of liquid nitrogen at the adsorption temperature). The hydrophobicity of samples was evaluated based on the surface water contact angles via a contact angle goniometer (SDC-200S, Dongguan city sindin Precision Instrument Co., Ltd., Dongguan, China). Fourier transform infrared (FT-IR) spectra of MCM-41, MCM-41-C_8_, CALB@MCM-41-C_8_, and free lipase CALB were determined using an FT-IR spectrometer (Bruker, Karlsruhe, Germany). Data from X-ray photoelectron spectroscopy (XPS) were obtained from the ESCALAB 250 XI (Thermo Fisher Scientific Inc., Waltham, MA, USA) device.

The protein content of lipase CALB before and after lipase immobilization was measured using the BCA method [42]. The loading amount and immobilization efficiency of the lipase were determined based on the calibration curve, which is prepared using the standard protein solution in the BCA kit with protein concentrations ranging from 0, 0.2, 0.4, 0.6, 0.8, and 1.0 mg/mL protein standard solution (Figure S1). 10 μL of the test sample was diluted and added to a clean 96-well plate, 10 μL of PBS buffer, and 200 μL of BCA working solution (50:1 homogeneous mix of solutions A and B) were placed in a 37 °C incubator for 15 min, and the absorbance of the enzyme solution was measured at a 562 nm wavelength in a microplate reader. The residual protein concentration in the lipase solution was calculated by:

(1) $$\mathrm{Loading}\;\mathrm{amount}\;(\mathrm{mg}/g)=\frac{{(C}_{0\;}-C)V}{M}$$

(2) $$\mathrm{Immobilization}\;\mathrm{efficiency}\;(\%)=\frac{C_{0}-C}{C_{0}}$$

where *C*_0_ and *C* are the protein concentrations (mg/mL) before and after lipase immobilization, respectively; *V* is the volume (mL) of the aqueous solution, and *M* is the mass (g) of MCM-41-C_8_.

### 2.4. Rapeseed Oil Deacidification Using CALB@MCM-41-C_8_

The enzymatic deacidification of high-acid rapeseed oil was achieved through the esterification between FFA and the acyl acceptor of glycerol catalyzed by CALB@MCM-41-C_8_. Firstly, 10 g of high-acid rapeseed oil was mixed with a certain amount of glycerol and a 4 Å molecular sieve at 45–85 °C, where glycerol was used as the acyl receptor and 4 Å molecular sieve could adsorb the water generated during the enzymatic esterification reaction. Then, the self-made CALB@MCM-41-C_8_ was added to the pre-heated solution to catalyze the esterification between FFA and glycerol. Meanwhile, 1–2 g of liquid sample solution from each reaction was taken at regular intervals for further analysis.

### 2.5. Oil Content and Composition Analysis by Gas Chromatograph

The contents and compositions of rapeseed oil were analyzed based on the method of Xu et al. [43]. The composition of the deacidification products was detected using a gas chromatograph (GC 2030, Shimadzu, Kyoto, Japan) equipped with a flame ionization detector and a fused silica capillary column (DB-5 HT, 15 m × 320 µm × 0.25 µm, Agilent Technologies, Santa Clara, CA, USA). The carrier gas was nitrogen, and the total gas flow rate was 2 mL/min. The injector and detector temperatures were maintained at 320 °C and 350 °C, respectively. The oven program started at 170 °C for 2 min; then, the temperature was raised at the rate of 5 °C/min until 380 °C and maintained at 380 °C for another 6 min. The split ratio was 50:1.

### 2.6. Oil Quality and Aroma Characteristics Analysis

The acid value and peroxide value of different oil samples were determined using the AOCS official methods Cd 3d-63 and Cd 8-53 [44]. Antioxidation of different oils including commercialized rapeseed oil, high-acid rapeseed oil, and deacidified rapeseed oil was analyzed using the oxidation inducer Rancimat (Model 743, Herison, Herisau, Switzerland), oil samples (3.0 g), including high-acid rapeseed oil, deacidified oil, and commercialized refined oil, which were heated at 110 °C under purified air at a flow rate of 20 L/h.

The concentration of volatile compounds was determined using the internal standard semi-quantitative method through solid phase microextraction (SPME) coupled with gas chromatography–mass spectrometry (GC–MS). Briefly, 5 g of oil sample was accurately weighed and mixed with 1 μL of 0.816 g/mL internal standard 2-methyl-3-heptanone at 50 °C for 20 min. Then, the volatile components were extracted with 1 cm 50/30 μm DVB/CAR/ PDMS fiber (Stableflex 24 Ga, Gray) for 30 min, which was injected into the gas chromatography injection port and desorbed at 250 °C for 5 min. The gas chromatograph analysis was carried out using an Agilent 7890A gas chromatograph on a DB-WAX (0.32 mm × 30 mm, 0.25 μm). The determination of volatile compounds in oil samples was based on the formula suggested by Yan et al. [45]:

(3) $$C_{i}=\frac{S_{i}}{S_{A}}\times C_{A}$$

where *C_i_* is the mass concentration of the target compound in μg/kg, *S_i_* is the mass concentration of the internal standard in rapeseed oil in μg/kg, *C_A_* is the peak area of the unknown compound, and *S_A_* is the peak area of the internal standard. In this experiment, the relative correction factor defaults to 1.

## 3. Results

### 3.1. Screening of Lipase and Mesoporous Molecular Sieves

**Figure 1:**
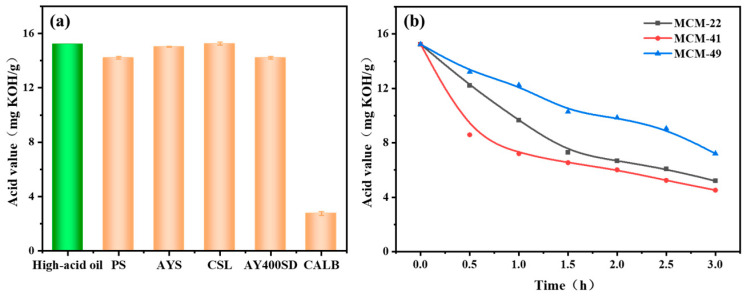
Screening of (**a**) different lipases and (**b**) carriers of mesoporous molecular sieves based on the enzymatic deacidification of rapeseed oil. The reactions were conducted in conditions with 10 g of high-acid rapeseed oil, 1 g of glycerol, 1 g of 4 Å molecular sieve, and 0.5 g of free enzyme/immobilized enzyme at 50 °C reaction temperature.

To identify the optimal free lipase and immobilized carriers, different free lipases including CALB, AYS, PS, CSL, AY400SD, and mesoporous molecular sieves (e.g., MCM-22, MCM-41, and MCM-49) were screened based on their performance for enzymatic deacidification. As shown in Figure 1a, compared to the free lipases such as AYS, PS, CSL, and AY400SD, which had little effect on enzymatic deacidification, lipase CALB effectively reduced the acid value of rapeseed oil from 15.2 mg KOH/g to 2.7 mg KOH/g in 3 h. The catalytic capacity of lipase CALB in oil deacidification was also confirmed by Xu et al. [43], who reported that the lipase CALB showed better performance in the enzymatic removal of FFA from high-acid rice bran oils, compared to the results achieved by other lipases such as Candida rugosa lipase, Lipozyme TL IM and Lipozyme RM IM [46]. The higher catalytic capacity of lipase CALB is mainly attributed to the serine residue in CALB, which can interact with the carbonyl group in FFA to form a complex between the acyl group and the enzyme, while the hydroxyl group in the glycerol will further react with the carbonyl group of the formed complex, leading to the re-release of serine residue for next round of reaction while the esterification reaction occurs [47,48]. Thus, lipase CALB is selected as the optimal biocatalyst for enzymatic deacidification for rapeseed oil. 

Moreover, the catalytic performance of immobilized CALB based on different mesoporous molecular sieves was also compared. It is noted that the acid values of rapeseed oils were gradually decreased from 15.25 mg KOH/g to 5.2 mg KOH/g, 4.52 mg KOH/g, 7.21 mg KOH/g by the immobilized lipase based on MCM-22, MCM-41 and MCM-49, respectively (Figure 1b). MCM-41 facilitated the immobilized CALB with higher deacidification, this is mainly related to its internal topology [49]. As shown in Table S1, the surface area of MCM-41 is 1439.3 m^2^/g, which is apparently higher than 440.5 m^2^/g and 1102.2 m^2^/g for MCM-22 and MCM-49, relatively. The higher surface area can promote lipase immobilization and subsequent contact between lipase and substrates [50]. Moreover, the proper pore size of 3.5 nm makes MCM-41 an ideal carrier to accommodate the lipase CALB and prevent it from leakage or damage during the stirring reaction. Hence, MSM-41 is screened and used for lipase CALB immobilization in the following experiments.

### 3.2. Characterization of CALB@MCM-41-C_8_

**Figure 2:**
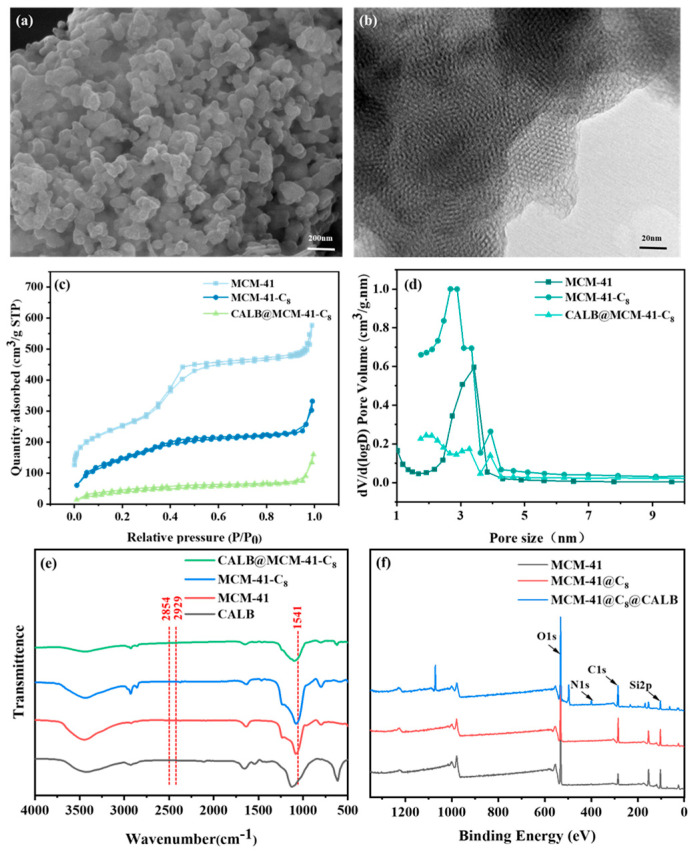
(**a**) SEM morphologies and (**b**) TEM image of MCM-41. (**c**) N_2_ adsorption–desorption isotherms and (**d**) pore size distribution curves, (**e**) FT-IR spectroscopy, and (**f**) XPS images of MCM-41, MCM-41-C_8_, CALB@MCM-41-C_8_.

The surface morphology and internal structures of MCM-41 were characterized using scanning electron microscopy (SEM) and transmission electron microscopy (TEM). The SEM results in Figure 2a show that MCM-41 molecular sieves are irregular and non-uniform spherical clusters, which is consistent with previous reports [51]. The TEM results in Figure 2b clearly indicate that MCM-41 were densely and orderly distributed hexagonal mesopores, presenting uniformly layered structures and fingerprint-like patterns. The nitrogen adsorption and desorption isotherms (Figure 2c) indicate that both MCM-41 and MCM-41-C_8_ showed type IV isotherms and H1 hysteresis loops with the properties of mesoporous materials [52]. The detailed text parameters obtained using BET analysis for MCM-41, MCM-41-C_8_, and CALB@MCM-41-C_8_ are presented in Table 1. It is evident that compared to MCM-41, the specific surface area of MCM-41-C_8_ decreased sharply from 1439.9 m^2^/g to 449 m^2^/g, indicating the effective grafting of C8 onto MCM-41. Additionally, the water contact angle of the MCM-41 was increased from 55° to 107° after surface modification (Figure S2), suggesting a shift towards a more hydrophobic carrier [53]. Similarly, the specific surface area of CALB@MCM-41-C_8_ was further decreased to 130.2 m^2^/g, along with the simultaneous reduction in pore volume from 1.0 cm^3^/g to 0.1 cm^3^/g. Hence, it is confirmed that lipase CALB has been successfully immobilized on the hydrophobic carrier of MCM-41-C_8_.

Furthermore, FT-IR spectroscopy was used to analyze the functional groups of the carrier and immobilized lipases. As depicted in Figure 2e, the characteristic peaks at 2929 cm^−1^ and 2854 cm^−1^ corresponded to the symmetric and asymmetric tensile vibrations of -C-H, respectively, suggesting the grafting of C_8_ onto MCM-41. Moreover, the characteristic peak at 1541 cm^−1^ in the spectrum of CALB@MCM-41-C_8_ refers to the -N vibration, thus confirming the immobilization of lipase CALB. Moreover, XPS further demonstrates the elemental peaks of C1s, Si2p, O1s, and N1s with a corresponding binding energy of 285.1 eV, 102.1 eV, 532.1 eV, and 399.1 eV, respectively (Figure 2f). Alternatively, compared to MCM-41, whose C 1s content was 16.3%, MCM-41-C_8_ and CALB@MCM-41-C_8_ showed a significant increase in C 1s content to 32.2% and 37.3%, respectively, as C8 modification increased the presence of carbon groups on carriers. Similarly, the N element yielded an increase from 1.1–1.3% to 4.1% due to the presence of amino groups in the immobilized lipases [54].

### 3.3. Optimization of Lipase Immobilization Conditions

**Figure 3:**
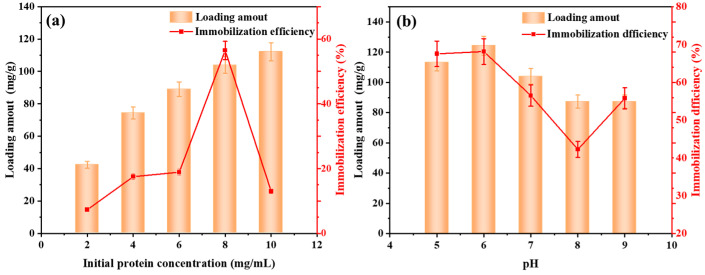
Effects of (**a**) initial protein concentration and (**b**) pH on the loading amount and immobilization efficiency of lipase CALB.

To increase the lipase loading and immobilization efficiency of CALB@MCM-41-C_8_, the key factors including the lipase solution and pH value of the phosphate buffer were investigated. As illustrated in Figure 3a, with the protein concentration continually increasing from 2 mg/mL to 10 mg/mL, the loading amount of lipase CALB was also increased from 42.3 mg/g to 112.2 mg/g, as increased protein concentration provided more lipase molecules to interact with MCM-41-C_8_, thus enhancing the lipase immobilization. Nevertheless, the maximum immobilization efficiency of 56.5% was noticed with the protein concentration reaching 8 mg/mL, while the further increase in protein concentration led to the significant reduction in immobilization efficiency to 13%. This can be explained by the saturation of lipase CALB on MCM-41-C_8_, as the increase in lipase loading amount became insignificant when the protein concentration reached 8 mg/mL while the further increase in protein concentration could not improve lipase loading but inversely decreased its immobilization rate [55]. Hence, the optimal protein concentration of 8 mg/mL is selected and used in the following study.

The influences of solution pH on lipase immobilization are illustrated in Figure 3b. The lipase loading was observed to slightly increase from 113.2 mg/g to 124.3 mg/g with the pH value increased from 5.0 to 6.0, followed by an unremitting decrease to 87.3 mg/g at pH 9.0. Similar results were also identified for the immobilization efficiency, which achieved a peak point of 68.2% at pH 6. The optimal value of pH 6 is mainly associated with the weakly acidic carrier of MCM-41, which requires the weak acid condition to keep its structural stability [56]. Hence, the optimum pH value of pH 6 is identified for the following lipase immobilization.

### 3.4. Optimization of Reaction Conditions

**Figure 4:**
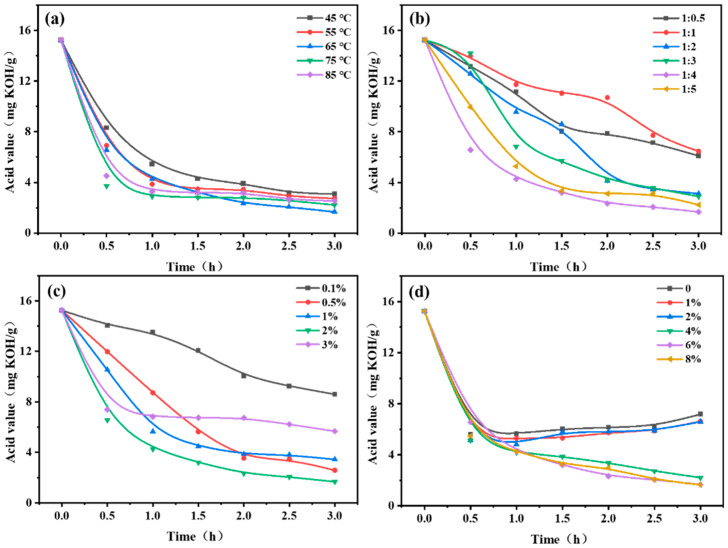
Effects of (**a**) temperature, (**b**) substrate molar ratio of FFA to glycerol, (**c**) adding an amount of CALB@MCM-41-C_8_, and (**d**) the addition of 4 Å molecular sieve on the enzymatic deacidification of rapeseed oils. Reactions were carried out in conditions with high-acid rapeseed oil (10 g), glycerol (0.02–0.1 mol), CALB@MCM-41-C_8_ (0.01–0.3 g), and 4 Å molecular sieve (0–0.8 g).

In this section, the reaction conditions such as reaction temperature, substrate molar ratio of FFA to glycerol, adding an amount of immobilized lipase, and 4 Å molecular sieve (water absorbent) were investigated based on the reduction of oil acid values. In the temperature range of 45–85 °C (Figure 4a), the acid value of rapeseed oil was drastically decreased during a 3 h reaction time. Specifically, the final acid value was reduced from 3.1 mg KOH/g to 1.7 mg KOH/g when the temperature was increased from 45 °C to 65 °C and further increased in temperature, leading to a final acid value rise to 2.2 mg KOH/g at 75 °C and 2.5 mg KOH/g at 85 °C. The first-half increase in temperature can not only promote the lipase activity but also minimize the system viscosity to enhance the lipase-substrate contract for improved enzymatic reaction [57]. However, a second-half temperature increase from 65 °C to 85 °C could reduce the catalytic ability of lipase CALB, thus leading to the mitigated performance in enzymatic deacidification [58,59]. Overall, lipase CALB presented the highest catalytic capacity at ~65 °C, and this is also consistent with the previous literature [60]. Hence, the optimal reaction temperature of 65 °C was determined for CALB@MCM-41-C8 in this study.

Glycerol is used as the acyl donor to facilitate the esterification with FFA, thus decreasing the acid value of rapeseed oil. The addition of glycerol significantly lowered down the acid values with the molar ratio of FFA to glycerol between 1:0.5 and 1:5 (Figure 4b). The lowest acid value of 1.7 mg KOH/g was from the reaction condition, where the molar ratio of FFA to glycerol was 1:4, suggesting that the inadequate or excessive amount of glycerol would diminish the enzymatic reactions. On the one hand, insufficient glycerol means limited FFA removal by the esterification between glycerol and FFA, leading to a relatively higher acid value. On the other hand, increasing the amount of glycerol can obviously boost the solution viscosity and delay the enzymatic catalysis. Moreover, the exceeded glycerol can partially cover the immobilized lipase to hinder the lipase–substrate contact and subsequently result in inefficient catalytic performance [61]. Thus, the optimal molar ratio of FFA to glycerol was identified to be 1:4.

In addition, at the optimal reaction temperature of 65 °C and a molar ratio of FFA to glycerol of 1:4, the effects of adding an amount of CALB@MCM-41-C_8_ on the enzymatic deacidification were studied. As depicted in Figure 4c, it was apparent that the use of 0.1–3% immobilized lipase promoted the removal of FFA. Particularly, increasing the adding amount of CALB@MCM-41-C_8_ from 0.1% to 2% further lowered the final acid value from 8.6 mg KOH/g to 1.7 mg KOH/g, thus demonstrating the catalytic effectiveness of the self-made immobilized lipase. However, the 3% of CALB@MCM-41-C_8_ addition conversely resulted in poor deacidification efficiency, reflected by the slightly higher acid value of 5.7 mg KOH/g. The excessive addition of immobilized lipase tended to agglomerate together to form larger clusters, which prevented the mass transfer and minimized the catalytic reaction [62]. Thus, 2% of CALB@MCM-41-C_8_ is regarded as the proper amount to add for the catalytic deacidification. 

To remove the byproduct of water generated during the esterified deacidification, a 4 Å molecular sieve worked as the water absorbent was used. Compared to the reaction without the use of water sorbent (namely, 0% of 4 Å molecular sieve addition), lower acid values were noticed with 1–8% addition of 4 Å molecular sieve, thus validating its feasibility in water removal and enhancement for enzymatic esterification (Figure 4d). Interestingly, 1–2% of 4 Å molecular sieve addition induced the acid value of rapeseed oil to significantly decrease from 15.3 mg KOH/g to 4.8 mg KOH/g in the first 1 h and inversely increased to 6.6 mg KOH/g at 3 h. This means that 1–2% of the 4 Å molecular sieve was insufficient and easily reached the saturation point in water absorption after 1 h of reaction, while the continually generated water during enzymatic esterification would cause the hydrolysis of TGA to produce more FFA and increased the acid value of oils [63]. Instead, with the addition of a 4 Å molecular sieve increased to 4–8%, the acid values were consistently reduced during the 3 h reaction to the lowest points at 2.2 mg KOH/g–1.7 mg KOH/g. Considering both the usage and cost, a 4% addition of 4 Å molecular sieve is selected as the optimal adding amount to remove the byproduct of water during the esterified deacidification.

### 3.5. Oil Quality and Aroma Characteristics Evaluation

#### 3.5.1. Lipid Composition in Rapeseed Oil

**Figure 5:**
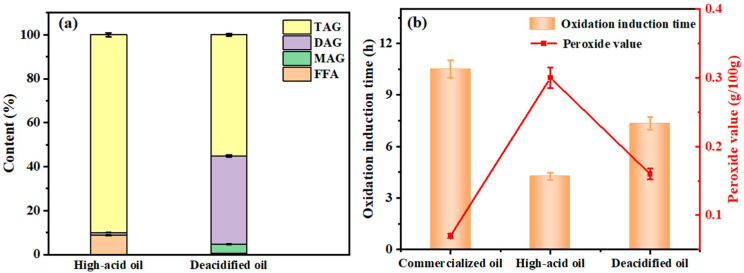
(**a**) Composition of lipid composition in rapeseed oil before and after enzymatic deacidification using CALB@MCM-41-C_8_; (**b**) antioxidant stability evaluation for commercial refined rapeseed oil and high-acid and deacidified rapeseed oils based on their oxidation induction time and peroxide values.

The main components such as TAG, DAG, MAG, and FFA in the rapeseed oils before and after enzymatic deacidification were compared. As shown in Figure 5a, the high-acid rapeseed oil consisted of 90.0% of TAG, 1.2% of DAG, 0 MAG, and 8.8% of FFA. After the enzymatic esterification catalyzed CALB@MCM-41-C_8_, both TAG and FFA were sharply reduced to 55.0% and 0.5%, respectively. Here, FFA reduction was associated with its esterification reaction with glycerol, while the decrease in TAG was mainly due to the glycerolysis reaction with glycerol and simultaneous TAG hydrolysis to some extent [64,65]. Such reactions could also promote the generation of DAG and MAG, which jumped to 40.2% and 4.3%, respectively. Both DAG and MAG are functional structural lipids that are widely used in food and healthcare areas [66,67]. Hence, enzymatic deacidification using glycerol as an acyl donor can not only effectively reduce the acid value of rapeseed oil but also simultaneously produce functional lipids to improve the oil quality economically and functionally.

Moreover, the antioxidant stability of deacidified rapeseed oil was compared with commercial refined rapeseed oil and high-acid rapeseed oil, based on the corresponding oxidation induction time and peroxide values (Figure 5b). Compared to the high-acid rapeseed oil, whose oxidation induction time was 4.25 h, the deacidified rapeseed oil and commercial refined oil extended the oxidation induction time to 7.35 h and 10.51 h, respectively. Namely, the deacidification to remove FFA is an essential strategy to improve the oil antioxidant stability [68]. This can be confirmed by the peroxide values, which were commercialized oil, high-acid oil, and deacidified oil for 0.07 g/100 g, 0.3 g/100 g, and 0.16 g/100 g, respectively. Since the peroxide values of both commercial oil and deacidified oil were below the standard level of 0.25 g/100 g for edible oil, it is concluded that enzymatic deacidification using CALB@MCM-41-C_8_ is applicable to improve the antioxidant stability of rapeseed oil based on the Chinese GB/T 1536-2021 standard [69].

#### 3.5.2. Analysis of Aroma Content in Rapeseed Oil

The aroma characteristics in the commercial refined rapeseed oil, high-acid rapeseed oil, and enzymatically deacidified rapeseed oil were identified based on the gas chromatography–mass spectrometry coupled (GC-MS, headspace solid-phase microextraction (HS-SPME) and NIST 17 database [70]. As shown in Table 2, a total of 38 aroma-active compounds were identified in three different rapeseed oils including aldehydes (5), sulfur-containing compounds (3), nitriles (9), heterocyclic compounds (8), acids (4), alcohols (4), ketones (2), and phenols (3). Specifically, 32 types of aroma-active compounds in commercial refined oil were detected with a total content of 47.1 μg/kg, in which nitrile compounds of 19.4 μg/kg and heterocyclic compounds of 10.6 μg/kg were the top two volatile compounds that occupied more than 63.7% of the total aroma contents, leading to the mixing aroma characteristics containing pickled vegetable, spicy, pungent, and roasted odors [71,72,73,74]. Compared to the high-acid oil with the main volatile compounds of nitriles (16.4 μg/kg) and aldehydes (5.2 μg/kg), the enzymatic deacidification by CALB@MCM-41-C8 led to higher aldehydes compounds of 37.7 μg/kg, thus altering the picked vegetable, spicy, and pungent aromas into fatty, bitter almond aromas. The increased aldehyde content in deacidified rapeseed oil could be related to the higher reaction temperature of 65 °C compared to the room temperature, as well as light exposure due to enzymatic reactions [75,76]. Overall changes were observed in volatile compound changes, thus validating the discernible impacts of different refining methods such as enzymatic deacidification on rapeseed oil’s odors.

### 3.6. Reusability and Applicability of CALB@MCM-41-C_8_

**Figure 6:**
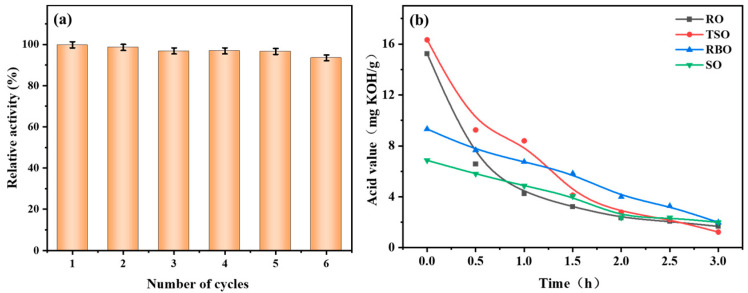
(**a**) Reusability and (**b**) applicability evaluation of CALB@MCM-41-C_8_. RO: rapeseed oil; TSO: high-acid value tea seed oil; RBO: high-acid value rice bran oil; SO: high-acid value soybean oil.

The reusability of CALB@MCM-41-C_8_ is an important index that is used to assess its long-term and economic benefits. The results show that the relative activity of CALB@MCM-41-C_8_ remained above 90% in six cycles, as the final acid values of high-acid rapeseed oil were kept below 3 mg KOH/g (Figure 6a). The findings confirm that MCM-41-C_8_ served as an ideal carrier to maintain the catalytic ability of lipase CALB and promote the utilization stability of immobilized lipase, thus demonstrating its potential implementation in industrial-scale applications. The broad applicability of CALB@MCM-41-C_8_ is studied based on its catalytic performance to deacidify various types of high-acid vegetable oils such as high-acid value tea seed oil (TSO), high-acid value rice bran oil (RBO), and high-acid value soybean oil (SO) (Figure 6b). With the use of CALB@MCM-41-C_8_ in optimal reaction conditions, the acid values of high-acid value tea seed oil, high-acid value rice bran oil and high-acid value soybean oil were continually decreased from 16.3 mg KOH/g, 9.3 mg KOH/g, and 6.9 mg KOH/g to 1.3 mg KOH/g, 1.9 mg KOH/g, and 2.0 mg KOH/g, respectively. Based on the results, the deacidified oils could meet the acid value demand of edible oil standard; therefore, CALB@MCM-41-C_8_ is applicable for enzymatic deacidification for different vegetable oils [77].

## 4. Conclusions

In this study, the immobilized lipase CALB@MCM-41-C_8_ was developed by immobilizing the lipase CALB on a hydrophobically modified mesoporous molecular sieve MCM-41 for the enzymatic deacidification of vegetable oils such as rapeseed oil, tea seed oil, rice bran oil, and soybean oil. The large surface area and suitable pore size of MCM-41-C_8_ due to its densely and orderly distributed hexagonal mesopores, the catalytic activity of immobilized lipase was significantly improved to facilitate the acid value of rapeseed oil to decrease from 15.2 mg KOH/g to 1.7 mg KOH/g, and DAG content was increased from 1.8% to 40.1% simultaneously. Moreover, the deacidified rapeseed oil also exhibited fatty, bitter almond aromas based on the aroma characteristics evaluation. To accelerate the industrial application of enzymatic deacidification, finding an easier method to separate immobilized lipase from the water absorbent of molecular sieves and high-yield enzymatic systems such as continuous-flow reactions would be a valuable future research direction. Overall, the self-made immobilized lipase CALB@MCM-41-C_8_ with good catalytic stability and broad applicability shows great potential in the greener processing of edible oils and cleaner production of functional lipids. 

## Figures and Tables

**Table 1:** Text parameters and elemental contents of sample characterization.

Sample	Text Properties			Elemental Content (Atomic%)			
	Surface Area (m^2^/g)	Pore Size (nm)	Pore Volume (cm^3^/g)	C1s	N1s	O1s	Si2p
MCM-41	1439.9	3.4	1.0	16.3	1.3	57.8	24.6
MCM-41-C_8_	449.0	4.1	0.5	32.3	1.1	45.3	21.4
CALB@MCM-41-C_8_	130.2	3.7	0.1	37.3	4.1	46.8	11.8

**Table 2:** The concentration of volatile compounds in different rapeseed oils.

Serial Number	Volatile Compounds	Odor Description	Aroma Content of Rapeseed Oil (μg/kg)		
			Commercialized Rapeseed Oil	High-Acid Rapeseed Oil	Deacidified Rapeseed Oil
**Aldehydes**					
1	Hexanal	Fatty, bitter almond aroma	1.34±0.02	1.44±0.05	2.13±0.04
2	Benzaldehyde		0.40±0.01	0.27±0.10	32.17±0.09
3	Heptanal		n.d.	0.41±0.01	0.17±0.05
4	Furfural		3.11±0.03	3.45±0.04	2.94±0.00
5	Benzeneacetaldehyde		0.15±0.07	0.17±0.06	0.22±0.07
**Subtotal**			5.00	5.74	37.63
**Sulfur-containing compounds**					
1	Dimethyl sulfide	Smoky aroma	0.32±0.02	0.08±0.05	0.19±0.19
2	Disulfide, dimethyl		0.18±0.03	0.11±0.01	n.d.
3	Dimethyl sulfone		0.09±0.05	0.05±0.03	n.d.
**Subtotal**			0.59	0.24	0.19
**Nitriles**					
1	2-Butenenitrile	Pickled vegetable aroma, Spicy and pungent	0.08±0.01	0.36±0.04	0.28±0.00
2	3-Butenenitrile		0.36±0.04	n.d.	0.06±0.00
3	4-Penenonitrile		7.27±0.03	0.54±0.05	n.d.
4	5-Hexenonitrile		7.31±0.02	12.23±0.00	n.d.
5	5-Methylthiopentonit-rile		0.03±0.04	n.d.	n.d.
6	Phenylpropionitrile		2.04±0.01	1.34±0.03	0.94±0.03
7	n-heptonitrile		n.d.	0.32±0.09	n.d.
8	(E, E)-2,4-pentadienorile		2.02±0.06	1.58±0.00	1.31±0.01
9	(E, Z)-2,4-pentadienorile		0.25±0.02	n.d.	n.d.
**Subtotal**			19.36	16.37	2.59
**Heterocyclics**					
1	2,5-Dimethylpyrazine	Roasted aroma	8.01±0.02	3.54±0.10	1.38±0.03
2	2-Amylfuran		n.d.	0.06±0.01	0.05±0.00
3	2,6-Dimethylpyrazine		0.04±0.02	n.d.	0.01±0.00
4	6-Methyl-2-ethylpyrazine		0.05±0.02	0.03±0.01	n.d.
5	5-Methyl-2-ethylpyrazine		1.49±0.04	0.88±0.03	n.d.
6	2,3,5-Trimethylpyrazine		0.61±0.05	0.38±0.02	0.04±0.03
7	2-Methyl-3,5-diethylpyrazine		0.25±0.10	0.36±0.05	0.02±0.01
8	2-Ethyl-3,5-dimethylpyrazine		0.16±0.04	0.10±0.04	0.02±0.00
**Subtotal**			10.61	5.29	1.52
**Acids**					
1	Acetic acid	Acerbity	7.00±1.00	3.39±0.08	0.32±0.03
2	Propionic acid		0.24±0.03	0.04±0.00	n.d.
3	Hexanoic acid		n.d.	2.32±0.03	n.d.
4	Pelargonic acid		n.d.	1.32±0.04	n.d.
**Subtotal**			7.24	8.07	0.32
**Ketones**					
1	5-Methylfuranone	Burnt, Meaty	0.43±0.01	0.14±0.01	n.d.
2	Furanone		0.17±0.17	0.14±0.04	0.09±0.00
**Subtotal**			0.6	0.28	0.09
**Alcohols**					
1	Isopropanol	Mellow, malty	0.53±0.09	n.d.	n.d.
2	Phenylethanol		0.15±0.01	0.02±0.00	n.d.
3	5-Methyl-2-furanoformaldehyde		0.94±0.04	0.28±0.03	0.20±0.04
4	Cyclobutanol		0.26±0.02	0.28±0.01	0.10±0.00
**Subtotal**			1.88	0.58	0.3
**Phenols**					
1	Y-butyrolactone	Creamy, sweet	1.76±0.07	1.24±0.01	0.65±0.03
2	4-Vinyl-2-methoxyphenol		0.08±0.03	0.10±0.04	n.d.
3	4-Vinyl-2,6-dimethoxyphenol		n.d.	1.73±0.00	1.10±0.01
**Subtotal**			1.84	3.07	1.75
**Total**			47.12	34.35	44.39

**Note:** n.d. represents the volatile compounds that cannot be detected in different oil samples.

## Data Availability

The original contributions presented in the study are included in the article/Supplementary Materials. Further inquiries can be directed to the corresponding authors.

## Supplementary Materials

The following supporting information can be downloaded at: https://www.mdpi.com/article/10.3390/foods13162539/s1, Table S1: Text parameters of different molecular sieves. Figure S1: Protein standard curve; Figure S2: Water contact angles of MCM-41 and MCM-41-C_8_.
