# Supplementary material for: Enzymatic Deacidification and Aroma Characteristics Analysis of Rapeseed Oil Using Self-Made Immobilized Lipase CALB@MCM-41-C8
Source: Foods. 2024 Aug 14;13(16):2539. doi: 10.3390/foods13162539 (PMC11353416; doi:10.3390/foods13162539)
Supplement: Supplementary file 1 [file foods-13-02539-s001.zip › foods-3110811-supplementary.pdf]

SUPPORTING INFORMATION

Enzymatic Deacidification and Aroma Characteristics Analysis of Rapeseed Oil Using Self-Made Immobilized Lipase CALB@MCM-41-C_8

Zhonghui Liu [1], Tieliang Liu [2], Run Liu [2], Qi Zhou [2], Yandaizi Zhou [1,*], Yi Zhang [2,*] and Mingming Zheng [2]

[1] Wuhan Institute of Technology, School of Chemical Engineering and Pharmacy, Key Laboratory of Green Chemical Process of Ministry of Education, Hubei Key Laboratory of Novel Reactor and Green Chemical Technology, Wuhan 430205, China; liuzhonghui0919@163.com

[2] Oil Crops Research Institute, Chinese Academy of Agricultural Sciences, Hubei Key Laboratory of Lipid Chemistry and Nutrition, Hubei Hongshan Laboratory, Key Laboratory of Oilseeds Processing, Ministry of Agriculture, Wuhan 430062, China; ltl13164666251@163.com (T.L.); liurun5764@163.com (R.L.); zhouqi01@caas.cn (Q.Z.); zhengmingming@caas.cn (M.Z.)

* Correspondence: ydzzhou@wit.edu.cn (Y.Z.); zhangyi07@caas.cn (Y.Z.)

Table S1. Text parameters of different molecular sieves

Figure S1. Protein standard curve

Figure S2. Water contact angles of MCM-41 and MCM-41-C_8

Table S1

Sample	Surface area (m^2/g)	Pore size (nm)	Pore volume (cm^3/g)
MCM-22	440.5	0.9	0.2
MCM-41	1439.9	3.5	1.0
MCM-49	1102.2	0.5	0.6

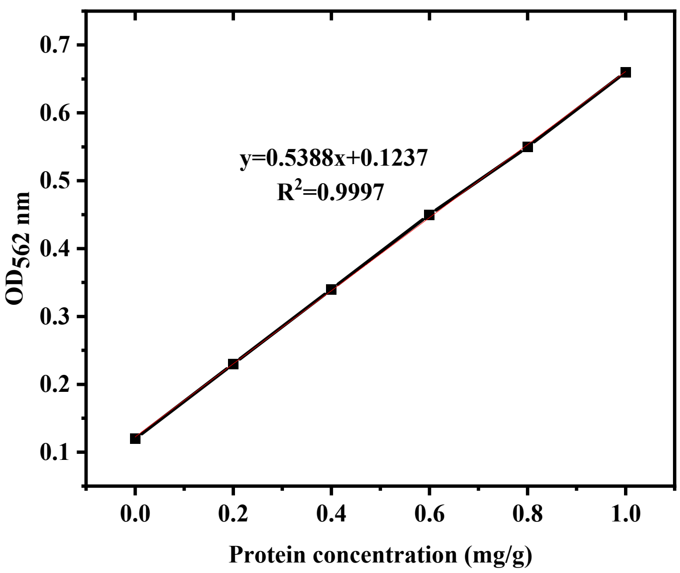

y=0.5388x+0.1237
R2=0.9997
OD562 nm
0.1
0.2
0.3
0.4
0.5
0.6
0.7
0.0
0.2
0.4
0.6
0.8
1.0
Protein concentration (mg/g)

Fig. S1

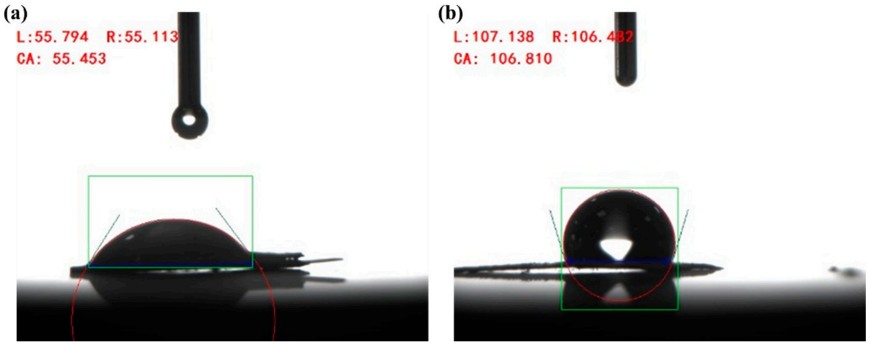

Fig. S2
